# Addressing opioid misuse through community-engaged strategy development: study protocol of a randomized controlled trial

**DOI:** 10.1186/s40900-024-00612-z

**Published:** 2024-07-19

**Authors:** Emily B. Zimmerman, Carlin Rafie, Sophie G. Wenzel, Kathryn Hosig, Domenique Villani, Jon Dance, Samantha S. Lee

**Affiliations:** 1 Center on Society and Health, Virginia Commonwealth University, Richmond, VA USA; 2 Department of Human Nutrition, Foods and Exercise, Virginia Polytechnic Institute and State University, Blacksburg, VA USA; 3 Department of Population Health Sciences, Virginia Polytechnic Institute and State University, Blacksburg, VA USA

## Abstract

**Background:**

Involving stakeholders in the research process facilitates collaboration, increasing understanding of factors influencing their wellbeing and motivating community action. Currently, there is a need for randomized controlled trials to evaluate the effectiveness of community-engaged research approaches for health, well-being, and engagement outcomes. In this study, we evaluate the effectiveness of both the SEED Method and a modified Delphi method in a participatory project to develop local strategies to address the opioid epidemic in three rural communities. The purpose of this study is to increase the level of evidence for community-engaged research methods through a randomized controlled trial.

**Methods:**

Two communities will use the SEED Method and one will use a modified Delphi method. We aim to recruit a total of 144 participants (48 per community). The evaluation team will randomize participants to an intervention group or a control group. In addition, we will collect outcome data from the participatory research team members leading the projects in each county (*n* = 18) and from additional community members who participate in focus groups (*n* = 32). The primary outcome for all participants will be the change in self-reported civic engagement as measured by the total score on the Individual Mobilization Scale.

**Discussion:**

In the context of participatory action to address opioid misuse in rural counties, this study will provide an understanding of the effectiveness of two community engagement methods for increasing civic engagement, as well as the extent to which participants successfully create locally tailored action strategies. The study will also explore how the observed effects differ depending on the participant’s role in the project (stakeholder participant, community research team member, or focus group participant), which is an important consideration for participatory research.

## Abstract (plain language summary)

Community-engaged research has become increasingly popular, yet there remains a need for randomized controlled trials that focus on testing the effectiveness of community-engaged research methods for outcomes relevant to the health, well-being, or civic engagement of individuals. Community members and researchers will benefit from data on the effectiveness of engagement methods. This will help them make decisions about which community engagement methods are best suited to project aims and participants’ expectations. This research protocol describes a study comparing the effectiveness of two community engagement methods, the SEED Method and the Delphi method, and a control intervention in three rural counties. In each of the counties, participants will be randomized to an intervention group using a community-engaged method or a control group using standard survey methods. Participants will create local strategies for addressing opioid misuse and prioritize those strategies for use in community action planning. For study purposes, we define opioid misuse as a problematic pattern of using opioids (prescription or illicit). Community members participating in the study represent a range of relevant stakeholders, such as people with Opioid Use Disorder, family members of people who use opioids, community service and health care providers, substance use treatment and recovery providers, and local policymakers. In each county, the research is being conducted by a diverse team of community members led by a Cooperative Extension member. We will assess changes in the self-rated civic engagement of participants and compare the number and types of strategies created and prioritized using the three methods evaluated.

## Background

### Problem definition

Stakeholders have a range of priorities and unique perspectives about the processes by which social and environmental factors affect community wellbeing and are increasingly finding opportunities to collaborate in research [1], ensuring that research priorities and community action reflect their concerns and preferences [2, 3]. Collaboration can improve equity and self-determination as well as research quality, validity, relevance, sustainability, and accountability, and it can deepen the understanding of the relationships among factors influencing community wellbeing and help drive community action [4–7].

The involvement of those impacted by health issues in the identification and prioritization of responsive strategies is important to ensure that the actions taken are based on an understanding of the local situation and reflect the communities’ concerns and needs [8]. Without community buy-in, many potentially effective strategies may not be achievable or useful strategies may be overlooked. Methods that successfully engage stakeholders tend to involve several key components: (1) guiding principles of engagement (e.g., trust, reciprocity, power sharing, co-learning), (2) structures and processes that encourage active participation and decision-making, and (3) capacity building so that every participant can leverage their expertise and learn new skills. A systematic process with clearly defined roles and activities can help provide these components while ensuring that stakeholders’ contributions are maximized, and project goals are met.

Community-based, participatory processes can contribute to the development of local strategies for addressing the causes and consequences of opioid misuse. Communities are faced with the high cost of health care, lost productivity, and escalating death rates due to prescription and illicit opioid misuse. Community input and collaboration are important to identify local needs and resources, communicate local values, find opportunities to build awareness, and develop programs and policies for prevention, treatment, and related services. In addition, at this time states, local governments, and tribes throughout the United States are seeking ways to identify local priorities to help allocate opioid abatement funds.

### Rationale of the study

There remains a critical need for evidence-based methods that facilitate engagement and capacity building throughout the research process [9–11] and facilitate collaborative research development by addressing issues such as tokenism, roles and power sharing that promote equity and the inclusion of underserved populations [12, 13]. Randomized trials have used community engagement methods in the service of testing an intervention, but the engagement method is rarely the intervention being tested. To the best of our knowledge, there have been a very limited number of randomized, controlled trials that focus on evaluating the effectiveness of community-engaged research methods for outcomes relevant to the health, well-being, or civic engagement of individuals. Russinova et al. (2014) evaluated the use of PhotoVoice combined with psychoeducation to reduce psychiatric stigma and found significant changes in measures such as self-stigma, coping, perceived recovery and growth, and community activism in the intervention group [14]. A systematic review of PhotoVoice research that examined the health effects of participation found eight randomized controlled trials (RCTs) and observed effects on health knowledge but not on health behavior or self-efficacy [15]. Thomas et al. conducted an RCT to evaluate the effectiveness of a deliberative method (community jury – an iterative process of education and deliberation) on knowledge of prostate-specific antigen screening and intention to screen [16]. Carman et al. conducted an RCT to evaluate the effectiveness of four public deliberation methods on knowledge of medical evidence and comparative effectiveness research and attitudes about the use of evidence in decision-making [17].

In previous research, we (EZ, CR) used the Stakeholder Engagement in Question Development (SEED) Method as a community-based participatory approach to involve local community members in developing strategies to address high rates of opioid misuse [18]. Participants were encouraged to create strategies covering any identified need (e.g., prevention, treatment, social determinants, research, policy). We have also invested in disseminating the SEED Method and building capacity for its use among researchers [19]. This study will test two participatory research methods (the SEED Method and a modified Delphi method) for their impact on improving civic engagement and quality of the project outcomes (strategies developed to address opioid misuse at the community level). This study leverages the presence of Cooperative Extension agents in three rural counties that are currently conducting opioid and substance misuse prevention to engage stakeholders within these communities in a process of co-education and analysis of the drivers of opioid and substance use, responsive strategy development, prioritization, action planning, and implementation. Virginia Cooperative Extension is a statewide education and outreach network that is part of the National Cooperative Extension System that uses research findings to equip Americans with the knowledge and skills to solve community problems. This research is set in rural communities due to the high opioid prescription and overdose mortality rates faced by many rural counties [20] and the specific challenges they face, including limited treatment availability, lack of workforce, and travel burden [21].

This study was designed by the academic and evaluation teams. Decisions regarding implementation, such as who will be on the community research teams, who to recruit as stakeholder and focus group participants, focus group topics, and use of data, will be made by the community research teams.

### Interventions

Two counties will use the SEED Method and one county will use a modified Delphi method. We were originally funded to implement the SEED Method in three counties but decided to implement the Delphi method in one county for comparative and logistical reasons.

### The SEED method

The SEED Method, developed at Virginia Commonwealth University (VCU) by Zimmerman and colleagues, was created to involve stakeholders at multiple levels in the research development process and is based on the principles of community-based participatory research (CBPR) [22]. It combines collaborative, participatory, and consultative engagement to provide meaningful participation from community members and other stakeholders. In this study, collaborative participation takes the form of community research teams that implement the SEED Method in their counties. Participatory engagement takes the form of specific stakeholder groups, known as *Topic groups*, that are selected based on their lived experience and professional expertise with the topic. Consultative engagement takes the form of participation in focus groups. Each of these three modes of engagement makes unique contributions to the project but work together iteratively. The SEED Method incorporates training and provides facilitation tools that lead the teams through the process of stakeholder selection, participatory conceptual modeling, and strategy or research question development and prioritization. Strategy development projects generally incorporate a phase of action planning and implementation.

The SEED Method has been used to develop stakeholder-generated research questions on diverse topics, including diet and behavioral management of diabetes and hypertension [23], lung cancer outcomes [24], knee surgery options [25], and health and homelessness [26]. Additional projects are underway on COVID-19, telehealth, cancer disparities, and developmental disabilities, among others. It has been used to develop strategies to address youth violence prevention and opioid misuse in both rural [18] and urban areas. The key potential benefits of the SEED Method include engaging a diverse set of stakeholders, incorporating community-based participatory research principles, having a specific engagement process that is laid out in steps and adaptable to different circumstances, providing tools and facilitation guides, facilitating activities in which groups of stakeholders develop their own models and strategies or questions, and building individual and community capacity. Participants in past projects indicated that the SEED Method prepares them well for project tasks and that they have a sense of satisfaction at gaining new skills in the process [27].

### The delphi method

Various versions and uses of the Delphi method have been developed since it was originally created by the RAND corporation in 1953 [28]. The Delphi method is widely used for engaging stakeholders, providing a systematic process through which stakeholders can share opinions on topic matters on which they have relevant expertise. While used in many different fields [29], it has been incorporated into various types of community-engaged research projects [30–35], commonly using purposive samples that bring together the views of more traditional subject experts and lay people. It is largely oriented to projects that require decision-making. Kezar and Maxey (2016) refer to the Delphi method used in participatory research as a means to address social needs as *change-oriented Delphi* [28]. The Delphi method involves a series of anonymous surveys that build toward attaining stakeholder consensus, although participatory projects may be less inclined to maintain anonymity throughout the process. Between surveys, participants usually receive the results of the previous survey. Subsequent surveys are more focused and build off of previous survey responses until consensus is reached. The strengths of the Delphi method include ease of implementation, its pragmatic approach, flexibility, and the incorporation of diverse stakeholders [36].

In sum, the SEED Method and the Delphi method are both used in community-based participatory research. The SEED Method provides a specific structure for engagement that can be adapted depending on the project. It can be conducted virtually or in person and relies heavily on discussion-based interaction. Main activities in the SEED Method include participatory modeling, strategy development, and strategy prioritization. Consensus is achieved through discussion, generally followed by voting. Including a large number of participants can be difficult because of the need to facilitate multiple groups, but additional perspectives can be incorporated through activities such as stakeholder focus groups and interviews. Implementation of the Delphi method in participatory research varies considerably and the most consistent component is the completion of a series of surveys completed by stakeholders, with feedback provided to participants between survey phases. Because the main activity is survey completion, it may be easier to add a larger number of participants. Consensus is reached through survey responses. Both methods can be used with a diverse mix of stakeholders, including those with lived experience.

### Methods and analysis

The aim of the proposed study is to increase the level of evidence for community-engaged research methods through a randomized controlled trial. The research objectives are to evaluate the effectiveness of the SEED Method and compare it to a modified Delphi method in opioid action planning projects in three rural Virginia communities.

### Study timeline

The intervention part of the study is planned to take place over about two years, with the first year devoted to implementing the intervention and the second year to implementing the strategies developed and prioritized by participants. The evaluation will take place over three years, allowing an additional year for follow up with participants, ending in Fall 2025.

### Research questions

Are there differences in outcomes between engagement approaches (SEED and Delphi)?

Are there differences between the intervention and control groups on the following key outcome variables?

1. Civic engagement (mobilization, motivation, and participation).
2. Strategy development (the number, type, and diversity of strategies created).

The hypothesis is that higher levels of engagement will result in greater change in civic engagement and greater diversity in proposed strategies.

### Outcomes measures

The primary outcome for all participants, civic engagement, will be measured as the total score on the Individual Mobilization Scale [37]. Mobilization refers to individual empowerment and human capital that facilitate contributions to community change. The Mobilization Scale, and the related Individual Community Related Empowerment Scale (ICRE), have been tested in several previous studies, [38, 39] and are being used as outcome measures of empowerment and social agency in additional protocols [40, 41]. This scale contains 24 questions in the categories of human capital, social assets, self-efficacy, motivation, and participation. The response categories for the questions are strongly disagree, disagree, not sure, agree, and strongly agree.

Secondary outcomes include Mobilization Scale subscores for motivation and participation, as well as key outcome indicators (number, types, and diversity of strategies developed) and process indicators. To collect these data, the pre- and post-surveys also cover demographics, group dynamics (e.g., perceptions that participants are able to speak openly and honestly, team members respect each other’s point of view, opinions are listened to and considered by other team members, all team members are made to feel welcome, and the team has been successful implementing project tasks), and group processes (e.g., satisfaction with meeting facilitation, how the team works, the way the team deals with problems, meeting location, meeting times, and compensation).

### Participants

Participants taking part in SEED Method Topic groups, the Delphi group, or control groups will be eligible if they live in the intervention counties or work on opioid-related issues in those counties, can participate in English, and are available to attend meetings as scheduled. Participants must meet additional eligibility criteria set by local teams in each county. These criteria aim to select stakeholders in high-priority groups, such as people who currently or formerly used opioids and their families, individuals who provide services to people who use opioids and people in recovery or treatment, and local decision makers (e.g., county officials, program managers, business and health system leaders). Local teams will decide the makeup of the groups and eligibility criteria. Each group may focus on one stakeholder type or may include a combination of several types of stakeholders. Participants will self-disclose whether they meet the eligibility criteria, and no formal screening will be employed. Because implementation in each county is conducted by the local community research team, they will decide in collaboration with stakeholders how and where to schedule meetings to accommodate participants’ needs and preferences and help ensure broad participation. For example, local teams will decide whether to schedule meetings for each group in person or virtually.

Additional participants include the community research team members and focus group participants. Community research team members are recruited by the Cooperative Extension agents leading project implementation in each of the three counties. Recruitment is based primarily on lived or professional experience with the topic. Focus groups are part of the SEED Method that are conducted to provide additional stakeholder perspectives to community research team members and Topic group stakeholder participants. The number and type of focus groups are based on the preferences of the community research teams and the Topic group members.

**Fig. 1:**
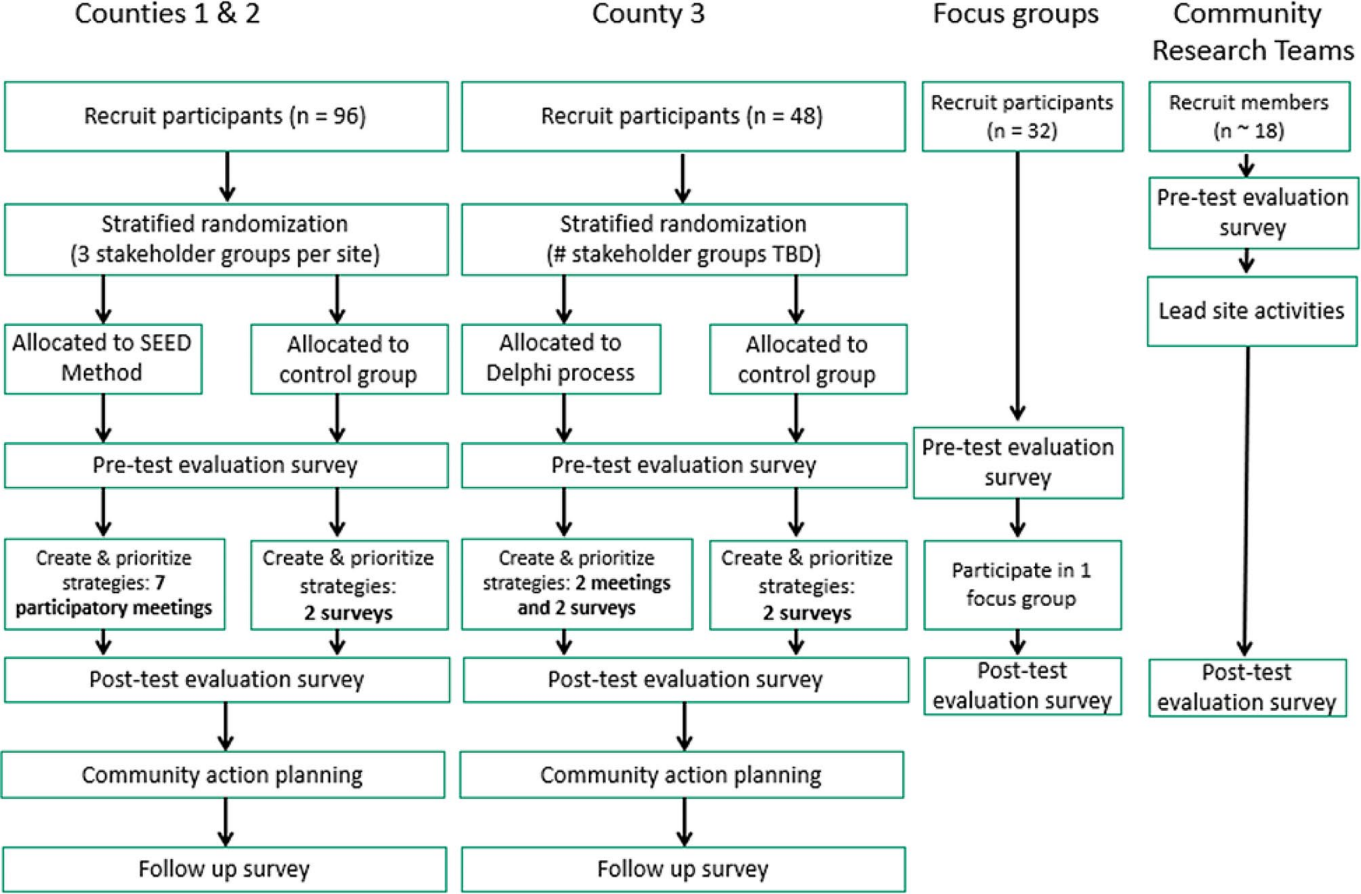
Study diagram: planned recruitment and activities

As illustrated in Fig. 1, the study will take place in Virginia counties located in southwest and central Virginia. The counties have lower median household incomes and educational attainment, higher poverty rates, and higher levels of disability than the state average. Each community is struggling to deal with a growing opioid and substance use problem and is experiencing higher than average substance and opioid overdose deaths. Like many rural communities, they have limited healthcare services. Cooperative Extension has a presence within each county, implementing an evidence-based youth and family substance prevention program. As shown in Table 1, the counties differ in racial and ethnic composition. Counties 1 and 2 are about one-third Black and county 3 is 94% White. Although they had similar rates of emergency room visits for opioid overdoses in 2022, the opioid overdose death rate in 2023 was 24 per 100,000 in county 1, compared to 17 per 100,000 in counties 2 and 3. The Neonatal Abstinence Syndrome rate varied considerably across the counties.

**Table 1:** County characteristics

	County 1	County 2	County 3
Population size 2022 (rounded)	34,000	22,000	30,000
Racial composition of the three largest population groups	White 60% Black 35% Multiracial 3%	White 61% Black 30% Multiracial 3%	White 94% Black 2% Multiracial 2%
Overdose ER visits 2022 (opioids only)	20 per 10,000	22 per 10,000	23 per 10,000
Overdose death rate 2023 (opioids only)	24 per 100,000	17 per 100,000	17 per 100,000
Neonatal Abstinence Syndrome cases per 1,000 births hospitalized	15 per 1,000	0 per 1,000	34 per 1,000

Sources: Population data: Data USA. (2022)

Opioid data: Virginia Department of Health. (2022). Emergency Department Visits. Retrieved from https://www.vdh.virginia.gov/drug-overdose-data/emergency-department/

Virginia Department of Health. (2023). Overdose Death Rates. Retrieved from https://www.vdh.virginia.gov/drug-overdose-data/overdose-deaths/

Virginia Department of Health. (2022). Neonatal Abstinence Syndrome (NAS). Retrieved from https://www.vdh.virginia.gov/drug-overdose-data/neonatal-abstinence-syndrome-nas/

### Participant compensation

Study participants are compensated according to their role. Cooperative Extension agents leading community interventions are paid as part of their salary. Other community research team members are paid an hourly wage. SEED and Delphi participants receive a stipend of $250, control group participants receive $70, and focus group participants receive $25. In addition, all participants receive compensation for each evaluation survey ($25) or interview ($50) they complete.

### Recruitment

Recruitment will occur through a combination of outreach strategies, including personal contacts, flyers and messages sent to partner organizations to be shared with potential participants, social media postings or advertisements, and advertisements in local media. The outreach materials will provide contact information for the study coordinator. The study coordinator will review eligibility criteria with potential participants and confirm eligibility for a specific stakeholder group within the relevant site. Once deemed eligible, the research coordinator will obtain informed consent. The target is 48 participants per county (144 total) divided as follows: 24 in the intervention group (For the SEED Method they are further divided into three stakeholder Topic groups) and 24 in the control group (composed of an equal number of stakeholders from each group). These are our target numbers, but flexibility will be allowed which may result in some stakeholder groups being larger or smaller than the target based on the community research teams’ ability to recruit.

### Randomization

A member of the evaluation team will randomize when there are eight consented participants in a specific group. Each of the eight consented participants will be assigned a unique number from one to eight. Using the “Sequence Generator” from random.org, participants will be randomized to an intervention or control group. This process will be repeated until each intervention group/control group has the target number of participants.

### Implementation

In each county, a local community research team will lead recruitment and activities. The composition of each community research team is planned as follows: one local Cooperative Extension agent, one project coordinator, and four additional community stakeholders.

In SEED Method counties, the community research teams will use the *SEED Stakeholder Identification Matrices* to identify three groups of stakeholders to engage in the process. The stakeholders are divided into separate groups primarily to emphasize their different perspectives and lived experiences, as well as to maximize affinity within the groups. The three Topic groups will attend seven meetings facilitated by their local community research teams. During these seven meetings, they will learn about the SEED Method process; review and discuss data about opioid use, opioid misuse, local statistics, and services; review focus group data from other stakeholders in their community; discuss relevant themes; engage in participatory conceptual modeling to explore potential causal factors and their interrelationships; participate in facilitated strategy development and strategy prioritization activities; and discuss community action planning. For each of the seven planned meetings, the Topic groups will meet separately; thus, each will develop their own conceptual models and list of prioritized strategies.

In the Delphi county, the community research team will identify which stakeholders to include in the intervention using discussion-based consensus. They will also finalize the contents of the surveys to be used to solicit information from participants via the Delphi method. Delphi participants will attend an introductory meeting facilitated by the local community research team to learn about the Delphi method; review and discuss data about opioids, opioid misuse, local statistics, and services. They will then receive an online survey asking them to identify strategies and a second online survey asking them to prioritize strategies. After each survey, they will receive a summary of the findings by email. The community research team will bring all Delphi participants together again to discuss the results and community action planning. All stakeholders will participate in the surveys and group meetings together, thus developing one set of strategies.

The control groups will be asked to develop a list of strategies like those developed in the intervention groups but without the participatory processes experienced in the SEED Method and Delphi groups. Control group participants in each county will receive two online surveys created by the evaluation team. The first survey will ask them to identify strategies, and the second survey will ask them to prioritize those strategies.

In sum, for the SEED Method, each Topic group will work independently and develop its own set of priority strategies, which will be combined for the action planning phase. For the Delphi method, all stakeholders will work together and develop one set of strategies. Control groups in each county will work separately, resulting in one set of strategies for each county.

### Evaluation activities

All research participants and community research team members will be included in the evaluation. Intervention and control group participants will be given an online pretest near the start of participation and an online posttest after activities are completed, as well as a follow-up survey approximately one year after the posttest (Table 2). Community research team members at the three sites will receive the same pre-, post-, and follow-up surveys. Focus group participants will receive a brief pre- and posttest survey only. For a subset of analyses, we will compare responses by participant type (intervention and control group participants, community research team members, and focus group participants). The evaluation surveys are distinct from the surveys taken by the Delphi method and control group participants. The former are designed to assess changes in key outcome measures (i.e., mobilization, motivation, and participation) to assess the impact of participatory research approaches on civic engagement, while the latter ask participants to propose and prioritize strategies to address opioid misuse in their communities.

**Table 2:** Evaluation data collection activities for RCT participants

	Pretest	Posttest	Follow-up
**Topic Group/** **Delphi Group – Intervention**	Demographic Survey Mobilization scale Community engagement survey Group dynamics (selected) Group readiness	Mobilization scale Community engagement survey Interview Group dynamics Process questions	Mobilization scale Community engagement survey Open ended responses
**Control**	Demographic Survey Mobilization scale Group readiness (selected)	Mobilization scale Process questions	Mobilization scale

A second outcome measure is strategy development (the number, type, and diversity of strategies created). To compare strategies across groups, members of the research team will code the strategies to indicate the focus, or domain, of each question. We will use the codes to calculate the number of domains addressed by each group and the number of domains (if any) that were unique to that group.

### Sample size and power analysis

**Table 3:** Number of planned participants by site and intervention type

	Site 1 (SEED Method)	Site 2 (SEED Method)	Site 3 (Delphi method)
**Intervention**	24 (3 groups of 8)	24 (3 groups of 8)	24 (1 group, with number of specific stakeholder groups TBD)
**Control**	24	24	24

The power analysis is calculated based on the sample sizes of the intervention and control group participants only. A target number of 48 participants per community will be recruited and randomized to the intervention or control condition (24 per condition) (Table 3). Minimal detectable differences between the variables of primary interest were estimated with this sample size, setting the alpha error at 0.05 and the beta error at 0.20, adjusting for the estimated ICC and the usual population variances based on cited research (Table 4). Based on these analyses, the anticipated sample size will have an 80% probability of detecting treatment differences in the primary outcomes if the true differences are in the range of the minimum detectable differences. These scores were calculated based on the standard deviations reported by Kasmel and Tanggaard [38].

**Table 4:** Minimum detectable differences (MDD) in primary outcome variables

Minimum Detectable Difference (MDD) and Percent Minimum Detectible Difference (%MDD)			
Estimated *n* = 144	$\:\underset{\_}{x}$ ^*^	SD^*^	MDD (%MDD)
Mobilization Scale (Total)	1.87	0.37	0.12 (6.5)
Participation Subscale	1.70	0.56	0.18 (10.9)
Motivation Subscale	1.83	0.56	0.18 (10.1)

### Data analysis

For the survey data, descriptive univariate analyses will be conducted on all study variables. The data will be checked for outliers, violations of normality and missing data. Predictors of drop-out and nonresponse will be explored to better understand any discernible systematic processes in play, taking advantage of the data collected until the last time point and demographic variables. If the missing data are found to be random and ignorable, multiple imputations will be used to address missing data [42]; otherwise, an intent-to-treat approach that makes full use of available data in determining intervention effects will be used for all analyses.

Scale scores will be calculated for all outcomes. The first step will be to calculate the intraclass correlation coefficient (ICC) to determine what percentage of the variance in the dependent variable is attributed to being from the same community. If the ICC at the community level is negligible, the community will not be used in the nested structure, and robust standard errors will be estimated to correct for the nested data structure. For primary and secondary outcomes, a two-level clustered longitudinal model with growth trajectories (change from Year 1 to Years 2 and 3) will be assessed at level 1 for each participant; variation in growth parameters among participants depending on the treatment status nested within location will be captured in the level 2 model. STATA will be used to estimate these two-level models. Covariates at different levels will be included at the two levels as predictors to isolate the effects on treatment outcomes. The two-level longitudinal model in STATA can be utilized for continuous as well as categorical and binary outcomes with minor changes in the interpretation of coefficients.

Data collected during the focus groups is for the purpose of including additional perspectives to the community research team members and Topic group members. Analysis of the data will focus on identifying themes from the information shared in the focus groups and summarizing them for discussion.

### Evaluator independence

An independent team from Virginia Tech’s Center for Public Health Practice and Research, separate from the academic team and community research teams, is responsible for the evaluation of the research project, including randomizing participants, collecting evaluation survey data, and assessing results. The evaluation team consists of a Principal Investigator trained in public health program evaluation, a project associate, and two graduate research assistants. All authors contributed to the evaluation plan, including team members responsible for planning and implementing the program. All team members will review and interpret the findings.

## Discussion

With the increasing popularity of community engaged research, there is an urgent need for data on the effectiveness of different engagement methods to help researchers and community partners make decisions about which methods are best suited to their needs. This research protocol describes a study comparing the effectiveness of two community engagement methods, the SEED Method and the Delphi method, and a control intervention. Participants in three rural counties will create local strategies for addressing opioid misuse and prioritize those strategies for use in community action planning. Community members participating in the study represent a range of relevant stakeholders. We will assess changes in the self-rated civic engagement of participants and compare the number and types of strategies created and prioritized using the three methods evaluated. This protocol describes a group randomized controlled trial to evaluate the effectiveness of these methods on increasing the civic engagement of participants and on the number, quality, and type of strategies created. Given the design of the study, we will be able to compare the SEED Method to the Delphi Method and compare both of these methods to the control group. We will also be able to explore how effects differed depending on participant role (community research team member, intervention/control group participant, and focus group participant).

The strengths of this protocol include replicating the implementation of the SEED Method, which was previously used in a rural Virginia county and a large midwestern city, to develop community strategies for reducing opioid misuse. Furthermore, we will use a rigorous randomized design with data collected at three time points. While the protocol was developed collaboratively with the previous developers of the SEED Method, an independent evaluation team will collect and analyze the data. Limitations could include the small sample size and the use of a relatively new outcome measure (the Mobilization Scale). Further limitations could result from the ability to recruit and retain the target number of participants in the rural communities. While we have specified recruitment targets and project activities, this is community-led research, and some decision-making will take place during implementation by community teams, which is considered a normal part of the process.

In addition to comparing community engagement methods, this project will highlight the ways in which community-based, participatory processes can contribute to the development of local strategies for addressing the causes and consequences of opioid misuse. Community input and collaboration are important to identify local needs and resources, communicate local values, find opportunities to build awareness, and develop programs and policies for prevention, treatment, and related services. The proposed strategies will be valuable to local governments seeking ways to identify local priorities to help allocate opioid abatement funds. Understanding the impact of the SEED and Delphi methods on strategy development may also help decision makers weigh potential community engagement options as they seek community input on opioid abatement priorities or other community needs.

## Data Availability

No datasets were generated or analysed during the current study.

## Abbreviations

CBPR: Community-based participatory research
ICC: Intraclass correlation
SEED: Stakeholder Engagement in Question Development
VCU: Virginia Commonwealth University
